# Comparison of Three International Definitions for Overweight and Obesity in a Population of Adolescents in Greece

**DOI:** 10.3390/jpm16050250

**Published:** 2026-05-03

**Authors:** Eleni M. Domouzoglou, Evangelia E. Ntzani, Michail I. Papafaklis, Anastasios Serbis, Ekaterini Siomou, Flora Bacopoulou, Assimina Galli-Tsinopoulou

**Affiliations:** 1 Pediatrics, Nafpaktos Health Center, 30300 Nafpaktos, Greece; 2 Program of Postgraduate Studies Adolescent Medicine and Adolescent Health Care, School of Medicine, Aristotle University of Thessaloniki, 54636 Thessaloniki, Greece; 3 Department of Hygiene and Epidemiology, Medical School, University of Ioannina, 45110 Ioannina, Greece; 4 Centre for Evidence Synthesis in Health, School of Public Health, Brown University, Providence, RI 02912, USA; 5 Biomedical Research Institute, Foundation for Research and Technology, 45110 Ioannina, Greece; 6 Medical School, University of Patras, 26504 Rio, Greece; 7 Department of Pediatrics, Medical School, University of Ioannina, 45110 Ioannina, Greece; 8 First Department of Pediatrics, Medical School, National and Kapodistrian University of Athens, Aghia Sophia Children’s Hospital, 11527 Athens, Greece; 9 2nd Department of Pediatrics, Faculty of Health Sciences, School of Medicine, Aristotle University of Thessaloniki, AHEPA University General Hospital, 54636 Thessaloniki, Greece

**Keywords:** obesity, overweight, adolescent, children, definition, WHO, CDC, IOTF

## Abstract

**Background:** Early diagnosis of obesity in adolescents is crucial for the prevention of severe health consequences. Different criteria for the diagnosis of obesity may lead to variations in prevalence. We aimed to compare the three most widely used international definitions (by WHO, IOTF and CDC) for overweight/obesity in adolescents in Northwestern Greece. **Methods:** A total of 403 adolescents aged 10–17 years were included. Agreement metrics and assessment of marginal heterogeneity and asymmetry were used for the comparison of the definitions. **Results:** In the total population, high agreement was observed among all definitions (Krippendorff’s alpha: 0.931, 95% CI 0.913–0.949). However, there was significant marginal heterogeneity (*p* < 0.001) and asymmetry (*p* < 0.001) for each pairwise comparison. WHO definition consistently yielded higher prevalence of obesity (WHO: 23.1%, IOTF: 15.4%, CDC: 21.6%). There were no significant differences in agreement (*p* = 0.247 for the comparison) between males and females, with more prominent marginal heterogeneity and asymmetry in males. Agreement among definitions was numerically lower in young adolescents aged < 14 years versus older ones (alpha: 0.919 vs. 0.953, *p* = 0.082), and systematic bias (*p* < 0.001) and asymmetry (*p* < 0.001) were present only in young adolescents without any significant difference in older ones. **Conclusions:** Although agreement was very high among definitions, they are not interchangeable, yielding different prevalence rates, particularly in young adolescents. IOTF criteria resulted in a reduced diagnosis of obesity and could lead to undertreatment; in contrast, WHO and CDC criteria may lead to overdiagnosis in our population. Caution is required when interpreting international criteria for overweight/obesity in various populations.

## 1. Introduction

Excess weight in children and adolescents, expressed as overweight and obesity, remains one of the most significant global epidemics [1]. Body mass index (BMI) has been defined to date as the best indicator of obesity in adults and children, but it has significant limitations. The most important limitation is that it is an indirect indicator of fat mass, in contrast to magnetic imaging and dual energy X-ray absorptiometry, which may be superior in this respect, but are less accessible in daily practice due to cost or availability [2]. Another very important factor affecting the accuracy of BMI in the assessment of obesity is its continuous change in relation to the child’s age, which has led the international scientific community to create percentile curves for BMI that can reflect this change. However, there is still no international consensus on the definition of obesity in children/adolescents. Achieving such an agreement would greatly help in understanding the causes of obesity and its prevention and treatment, as it would allow the evaluation and direct comparison of the results of international studies. Currently, there are mainly three different definitions from three major international health organizations: the World Health Organization (WHO), the International Obesity Task Force (IOTF) and the United States Centre for Disease Control and Prevention (CDC) [3,4,5]. All three of them are the most frequently used definitions worldwide to assess childhood obesity rates in various studies in different countries and meta-analyses. However, it is not clear whether these definitions, when applied to different ethnicities, under- or over-diagnose any of the phenotypes (obese, overweight, normal weight) [6,7,8]. There is a need to establish precise cutoff values for the diagnosis of obesity tailored to individuals of different age groups, geopolitical and socio-economic conditions, that would enable effective prognosis and treatment.

National reference charts would ideally assess the obesity rates of respective populations. Interestingly, differences arise from measuring BMI using national and international standards. This observation comes from various countries, including Greece. In 2011, a study of 1557 boys and girls aged 11.42 ± 3.51 years old from Thessaloniki showed an increased number of children in the overweight and obesity groups identified by using the IOTF and CDC definitions of obesity compared to those identified by using the national definition [9]. In a study of 258 children aged 3 to 6 years in Northeastern Italy, BMI was measured, and the results for overweight/obesity were compared based on three different definitions: CDC, IOTF and the national definition. The assessment of overweight was similar for all three definitions, but the assessment of obesity using the CDC definition led to significantly higher prevalence in both sexes compared to the other two definitions [10].

In Greece, the definition based on the WHO assessment is frequently recommended, while growth curves derived from this definition are distributed in the Personal Child Health Record. The importance of national growth curves remains significant because the characteristics of a population, such as average final height and average weight, vary significantly between countries depending on the socio-economic, genetic and health-policy characteristics [11]. Based on this observation, local weight, height and BMI curves may be more representative of the average child and adolescent living at a given time in a given country. Such data contradict the need to broaden our understanding of obesity at an international level, which would require common measures of comparison between populations, rather than local growth curves [12]. For these reasons, understanding obesity is a very complex issue, and conclusions drawn from different studies should take into account the definition criteria used and the population in which they were conducted. A recent systematic review and meta-analysis highlights the importance of defining a gold standard for the assessment of overweight and obesity in children, which would ideally integrate information about the nationality, the geographic region and the secular growth [13]. The authors conclude that the great heterogeneity that arises in studies from different ethnicities indicates the practical difficulties and the magnitude of the problem. Data on the application of all three international definitions in a Greek population of adolescents are not yet available.

The aim of the present study was to compare the agreement among the three above-mentioned definitions and investigate any heterogeneity regarding the prevalence of overweight and obesity in a population of adolescents in Northwestern Greece.

## 2. Materials and Methods

This is a retrospective study of adolescents aged 10–17 years who attended the outpatient department of Pediatrics (University Hospital of Ioannina) for routine clinical examination either for sports reasons or for follow-up after recent hospitalization for mild symptoms that do not affect growth and body weight. These spanned from Jan 2012 to May 2022. These adolescents came from the wider region of Epirus in Greece. The study was conducted in accordance with the Declaration of Helsinki, and approved by the Ethics Committee of the University Hospital of Ioannina. 

The study included anthropometric measurements from 403 adolescents of both sexes aged 10 to 17 years. Weight was measured on a precision electronic scale and height on a wall-mounted stadiometer, after at least 6 h of fasting, with light clothing and without shoes. BMI was calculated using the Quetelet formula: body weight (kg)/[body height (m)]^2^. Overweight/obesity in adolescents was diagnosed based on the three international definitions: WHO, IOTF and CDC [14]. The WHO definition (2006) diagnoses overweight as a BMI ≥ 1 standard deviation (SD) and ≤2SD above the normal average, and obesity as a BMI ≥ 2SD above the normal average. For ages 5 years and under, it was based on data from a multicentre international study (Multicentre Growth Reference Study, MGRS) conducted between 1997 and 2003 in certain areas of Brazil, Ghana, Norway, Oman and the United States of America under selected socio-economic conditions that promote infant growth, and were considered ideal conditions. For ages 5 to 19, it was based on data collected during the NHES II, III and NHANES I studies, ensuring a smooth transition from the pre-existing curves for ages under 5 years [14]. The IOTF definition was published in 2000 and is based on BMI curves that extend the adult limits of 25 kg/m^2^ for overweight and 30 kg/m^2^ for obesity to the age of 18 years. It was based on data collected between 1963 and 1993 in six different countries: Brazil, the United Kingdom, Hong Kong, the Netherlands, Singapore and the United States of America. The CDC definition diagnoses overweight for a BMI ≥ 85th percentile and obesity for a BMI ≥ 95th percentile and was developed in 2000 based on data collected between 1963 and 1994 in the United States during the large National Health Examination Surveys (NHES) II, III and National Health and Nutrition Examination Surveys (NHANES) I, II and III.

### Statistics

Continuous variables are presented as median (interquartile range) and categorical variables as count (percentage). Agreement between classification definitions for the three weight categories (normal weight, overweight, obesity) was evaluated using both overall and pairwise measures. Overall agreement across all three definitions was quantified using Krippendorff’s alpha, and pairwise agreement between definitions was assessed using weighted Cohen’s kappa, both applying quadratic weights to account for the ordinal nature of the weight categories. Power considerations were based on simulation-based guidelines for agreement coefficients [15,16]. With three definitions and three ordinal weight categories, the sample of 403 adolescents provided very high power (>99%) to distinguish a hypothesized “excellent” agreement level (Krippendorff’s alpha ≥ 0.90) from the standard reliability threshold of 0.80.

Differences in the distribution of adolescents across the three ordinal categories (normal, overweight, obesity) were assessed for marginal homogeneity using the Stuart–Maxwell test, which was performed pairwise for each combination of definitions to identify systematic shifts in classification. Additionally, an exact test of symmetry, which ensures validity regardless of cell frequencies, was used to evaluate the symmetry of disagreements within each pairwise contingency table.

Additional stratified analyses were performed to evaluate potential bias across gender and age groups and detect potential group-specific shifts in classification prevalence between definitions. To determine if the level of agreement differed significantly between groups, comparisons of quadratic-weighted Krippendorff’s alphas were performed between gender (males vs. females) and age (<14 years vs. ≥14 years) subgroups; the rationale for the age subgroups is based on the fact that in the large majority of children/adolescents puberty onset and rapid growth spurt (including peak height velocity) have occurred by the age of 14 years. These comparisons were conducted using a Z-test for independent agreement coefficients.

The *p*-values for individual pairwise comparisons (using the Stuart–Maxwell test and the exact test of symmetry) between the different definitions were adjusted, taking into account multiple comparisons (i.e., three pairwise comparisons for the three definitions) using the Bonferroni method (multiplication of the *p*-value). The level of nominal statistical significance was set at a *p*-value less than 0.05. The Statistical Package for Social Sciences (version 26.0 for Windows, SPSS Inc., Chicago, IL, USA) and Stata-Statistical software for data science (version 17.0 for Windows, StataCorp LLC, College Station, TX, USA) were used for statistical analysis.

## 3. Results

The primary descriptive statistics of our population (403 adolescents) are included in Table 1. There was a higher representation of males (54.8% of the total population). No statistically significant differences were observed in age, weight, height and BMI between males and females.

**Figure 1:**
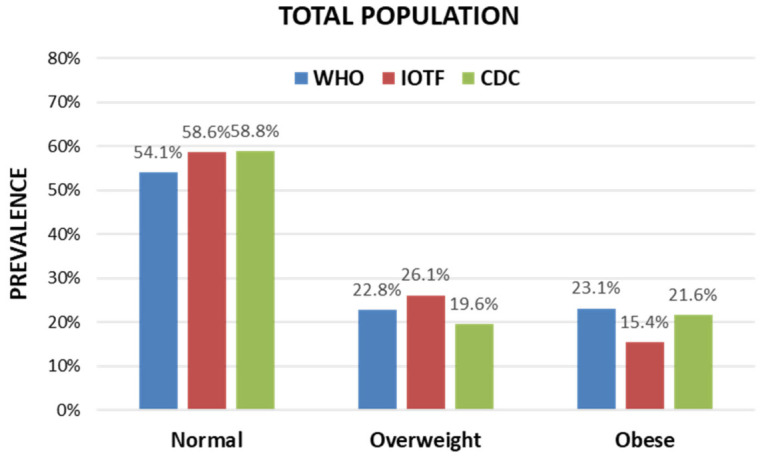
Comparison of the prevalence of the three weight categories according to the three international definitions (WHO, IOTF, CDC) in the total population. There was significant marginal heterogeneity (*p* < 0.001) with high asymmetry (*p* < 0.001) for all pairwise comparisons between the definitions.

In the total population, the results of cross-tabulations comparing the three definitions in pairs are presented in Table 2. There was high agreement among the three definitions (Krippendorff’s alpha: 0.931; 95% CI 0.913–0.949) with high pairwise weighted kappa values (WHO vs. CDC: 0.950; WHO vs. IOTF: 0.902; CDC vs. IOTF: 0.939). However, despite this strong agreement, the Stuart–Maxwell test revealed significant marginal heterogeneity (*p* < 0.001 for each pairwise comparison after applying Bonferroni correction), indicating a systematic bias between the classification definitions, and the pattern of disagreements was highly asymmetrical (exact test: *p* < 0.001 for each pairwise comparison after applying Bonferroni correction). Figure 1 shows the prevalence of each weight category according to the definitions, indicating that the WHO definition is “stricter”, consistently yielding a higher classification of obesity (WHO: 23.1%, IOTF: 15.4%, CDC: 21.6%) and a lower classification of normal weight. Also, Figure 1 demonstrates that the IOTF definition primarily yields lower classification of obesity with higher classification of overweight.

### 3.1. Subgroup Analysis in Males and Females

Considering the two sexes separately (cross-tabulations in Table 3 and Table 4 for males and females, respectively), the agreement was overall high in both of them (Krippendorff’s alpha: 0.922, 95% CI 0.897–0.946 for males vs. 0.943, 95% CI 0.917–0.969 for females; Z-statistic for the comparison: *p* = 0.247) with numerically lower pairwise weighted kappa values in males (WHO vs. CDC: 0.958; WHO vs. IOTF: 0.883; CDC vs. IOTF: 0.920) than in females (WHO vs. CDC: 0.939; WHO vs. IOTF: 0.927; CDC vs. IOTF: 0.963).

**Figure 2:**
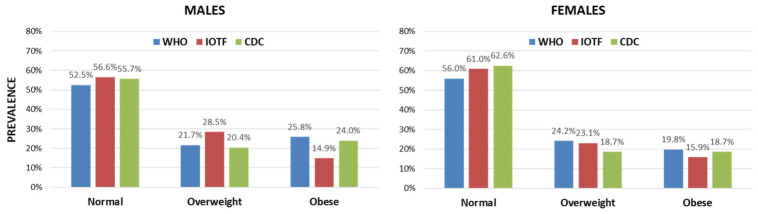
Comparison of the prevalence of the three weight categories according to the three international definitions (WHO, IOTF, CDC) separately for males and females. There was significant marginal heterogeneity (*p* < 0.05) with high asymmetry (*p* < 0.05) for all pairwise comparisons between the definitions except for the comparison of CDC vs. IOTF in females (*p* = 0.055 for marginal heterogeneity and *p* = 0.047 for asymmetry).

However, in a similar fashion to the total population, the definitions resulted in differences in prevalence rates with significant high marginal heterogeneity and an asymmetric pattern of disagreements in the majority of pairwise comparisons (Stuart–Maxwell: *p* < 0.05 and exact test of asymmetry: *p* < 0.05) except for the comparison of CDC vs. IOTF in females (Stuart–Maxwell: *p* = 0.055 and exact test of asymmetry: *p* = 0.047). Figure 2 demonstrates the prevalence of the weight categories according to the definitions separately for males and females. The WHO definition again yielded higher classification of obesity while the IOTF one resulted in the lowest classification of obesity and the highest of overweight; these differences were more evident in males (obesity: WHO 25.8%, IOTF: 14.9%, CDC: 24.0%; overweight: WHO 21.7%, IOTF: 28.5%, CDC: 20.4%) than in females (obesity: WHO 19.8%, IOTF: 15.9%, CDC: 18.7%).

### 3.2. Subgroup Analysis in Younger (<14 Years) and Older (>14 Years) Adolescents

**Figure 3:**
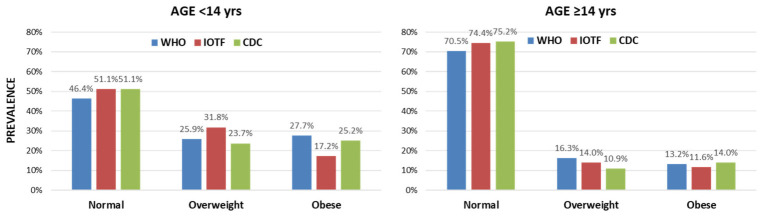
Comparison of the prevalence of the three weight categories based on the three international definitions (WHO, IOTF, CDC) separately for adolescents aged below and ≥14 years. Significant marginal heterogeneity (*p* < 0.001) with high asymmetry (*p* < 0.001) was observed for all pairwise comparisons between the definitions only in the younger group. In the older group, there was no systematic bias or asymmetry (*p* > 0.05 for both).

We also aimed to assess our population taking into account the onset of puberty and the growth spurt (including the age of peak height velocity) that takes place within approximately two years after puberty onset, in younger adolescents aged < 14 years old [17]. For this reason, we grouped our population into younger (<14 years old) and older (≥14 years old) adolescents (Figure 3 and Table 5 and Table 6). From this analysis, a high overall agreement was observed in both groups, which was more pronounced in the older group (Krippendorff’s alpha: 0.919, 95% CI 0.896–0.942 for younger vs. 0.953, 95% CI 0.922–0.985 for older adolescents; Z-statistic for the comparison: *p* = 0.082). Accordingly, all pairwise weighted kappa values were above 0.94 in the older group (WHO vs. CDC: 0.947; WHO vs. IOTF: 0.944; CDC vs. IOTF: 0.968) but numerically lower in the younger group (WHO vs. CDC: 0.948; WHO vs. IOTF: 0.882; CDC vs. IOTF: 0.925).

Figure 3 demonstrates the prevalence of the weight categories according to the definitions separately for the two age groups. Overall, the prevalence rates of overweight and obesity according to all definitions were reduced in the older group. Differences in the prevalence rates were prominent in the younger group with significant systematic bias (*p* < 0.001) and asymmetry (*p* < 0.001) for all pairwise comparisons between definitions. In contrast, no significant systematic bias (WHO vs. CDC: *p* = 0.091; WHO vs. IOTF: 0.091; CDC vs. IOTF: *p* = 0.406) or asymmetry (WHO vs. CDC: *p* = 0.094; WHO vs. IOTF: 0.094; CDC vs. IOTF: *p* = 0.75) was observed in older adolescents.

## 4. Discussion

This is the first study in a Greek population to compare all three major international definitions in adolescents. The results of our study add knowledge regarding the application and performance of the three international definitions in adolescents coming from Northwestern Greece. Our main findings demonstrate a high overall agreement among the three commonly used overweight/obesity definitions in adolescents. However, there appears to be systematic bias with classification shift for the diagnosis of overweight and obesity according to these three definitions, thereby indicating that they cannot be used interchangeably. The detailed comparison of the prevalences of overweight/obesity shows that the WHO definition classifies adolescents with obesity with the highest rate, and the IOTF definition leads to the lowest rates of obesity, with a concomitant higher rate of overweight diagnosis. Similar results were observed in both sexes, whereas the above-mentioned significant systematic bias was only found for younger adolescents and not for older ones who have completed most of their growth spurt trajectory.

It is worthwhile noting that a rather high prevalence of overweight/obesity was observed in the adolescent population studied, irrespective of the definition used, and there appears to be a worrying increase compared to previously published studies presenting data in the same region. In 2016, the prevalence of overweight/obesity based on the IOTF definition among 13-year-old adolescents in Greece was studied and a map was created showing the regional results; prevalence rates of 19% for overweight and 7.9% obesity in 13-year-old males, and 28.6% and 6.3%, respectively, in females of the same age were reported in Epirus [18]. Although our results are not directly comparable because they cover a much wider age range, our study shows an increase of approximately 50% in the prevalence of overweight in males (28.5%) and an almost doubling of the prevalence of obesity based on the same definition (14.9% in males and 15.9% in females). In the EYZHN study published in 2018, 336,014 children and adolescents aged 4 to 17 from all over Greece were studied, and the prevalence of obesity/overweight was measured based on the IOTF definition [19]. The prevalence of overweight and obesity in the total population studied was 22.2% and 9% for males and 21.6% and 7.5% for females, respectively. These results are similar to our findings for overweight, but are almost half of the obesity rates compared to our study. The EYZHN study also observed a decrease in the prevalence of overweight/obesity in adolescents over 12 years of age compared to the rest of the population studied, aged 4 to 11 years. The GRECO study included 4786 children aged 10 to 12 years from 10 different regions of Greece (one of which was Northwestern Greece) and measured the prevalence of overweight/obesity based on the IOTF definition [20]. The results showed a prevalence of overweight/obesity of 29.9%/12.9% for males and 29.2%/10.6% for females, respectively. These results are similar to the results from our population.

Evidence from the literature underlines the risk of underestimating obesity and overestimating overweight rates in adolescents by using the IOTF definition, and the converse possibility of overestimating obesity by using the WHO definition, as stated in an Iranian and a Korean study [21,22]. In both these studies, national cut-points were also taken into account and served as baseline measurements representing each population. A previous study in Northern Greece assessed all three stated international definitions, but the children included were preschoolers, 2–6 years old. They concluded that diagnoses vary significantly based on the criteria used, with the CDC definition scoring the highest rates of children with overweight, including obesity (i.e., considered as one broad group) in comparison to the other two definitions [23]. A recent large-scale epidemiological study in Western Greece used two of the definitions (CDC and IOTF) for diagnosing overweight/obesity in adolescents aged 10–16 years old, and they described a lower prevalence of obesity by the IOTF in comparison to the CDC criteria, with similar rates of overweight by age and sex [24]. Our study raises similar concerns, showing that the IOTF definition, compared to the CDC, diagnoses fewer adolescents with obesity while they identify approximately the same number of adolescents with normal weight (Figure 1 and Table 2). In the present study, the significantly fewer adolescents with obesity according to the IOTF definition is due to a higher diagnosis of adolescents with overweight (Figure 1 and Table 2). The WHO definition identifies fewer adolescents with normal weight compared to the IOTF, while among those with increased weight, the number in the overweight group is almost equal to that in the obesity group. Our observations were comparable between boys and girls.

We speculate on the possibility of over- or underdiagnosing overweight/obesity by using each of the three definitions we assessed; it is likely that for every possible case of overdiagnosis, another possible case of underdiagnosis exists. The distinction between normal weight and overweight/obesity is important prognostically and may trigger therapeutic decisions. As an adjunct to initial lifestyle intervention, pharmacotherapy may be offered to every child above 12 years of age with obesity after evaluation of risk and benefits, based on the recent guidelines by the American Academy of Pediatrics [25]. Such recommendations for potential advanced interventions warrant additional caution to avoid the risk of overtreating individuals. Conversely, in the case of underdiagnosis, an opportunity to adopt healthier behaviours may be lost. Failure to diagnose obesity would lead to higher rates of untreated adolescents with obesity who are predisposed to multiple metabolic and cardiovascular disorders in adulthood. The current findings, along with the previous literature data, are alarming and highlight the need for precise and tailored tools for the diagnosis of overweight/obesity in children and adolescents. In Greece and more specifically in our population characterized by high overall rates of obesity, it would be of utmost importance to accurately identify adolescents requiring appropriate management. Such attention would include interventions on daily activity and nutrition at home and at school, national awareness by public policies and dedicated informative campaigns in mass media, as well as health care providers’ training. An important initiative for the appropriate training of Pediatricians and General Practitioners has been developed in Greece by the “National Registry for the Prevention and Management of Overweight and Obesity in Childhood and Adolescence”, supported by interoperability with other national infrastructures [26]. Efforts are also being made to approach the matter by applying screening tools for diagnosing overweight/obesity, such as the CORE index, which is proposed for early prediction of the condition using anthropometric and socio-demographic data. It is based on analysis from a representative sample of 5946 children and adolescents [27]. This index is calculated based on the IOTF definition for childhood obesity; however, it represents a good example of how information from socio-demographic data and anthropometric measures may be combined to increase precision in the diagnostic process. These actions, combined with further research based on Nationwide data, where national cut-off values for BMI categories may serve as a baseline, are important to create accurate tools.

A different aspect of misdiagnosis of the weight category is the overdiagnosis of overweight, which, as mentioned in the literature, carries the risk of identifying as overweight those individuals whose physical measurements (height and weight) align with the upper percentile ranges for their age and gender [28]. This was also evident in a recent Australian study of approximately 4500 children aged 8 to 12 years, which described how increasing lean body mass and height can influence and lead to overdiagnosis of overweight and obesity using the IOTF definition. For this reason, it is recommended that BMI be considered in conjunction with the height percentile [28]. The same study demonstrated that the age at which height has the greatest impact on BMI is between 8 and 12 years, which is directly linked to the onset of puberty. These observations make it necessary to take into account the onset of puberty at 8–12 years when assessing obesity rates in adolescents. The range of the IOTF definition of overweight in these age groups is wider and is related to the fact that the difference in body measurements between peers varies considerably depending on the stage of puberty. In that respect, the WHO provides graphs comparing BMI percentile curves based on the WHO and IOTF definitions for males and females separately [29]. These graphs show that the BMI cutoffs according to the IOTF, mainly for the diagnosis of obesity but also for overweight, are shifted higher on the BMI percentile curves, primarily for the ages 8 to 14 years. As a result, more children in these age groups meet criteria for overweight and fewer for obesity compared to the WHO diagnosis. Consequently, it would be reasonable to subdivide adolescents into group ages younger and older than 14 years, based on the stage of puberty onset/growth spurt, and taking into account the fact that the differences between the definitions are smoother after the age of 14 years, as indicated in the comparison of the percentiles provided by WHO. Accordingly, we grouped our population into adolescents aged < 14 years and ≥14 years. It was observed that for ages ≥ 14 years, there was no significant difference in the classification of overweight/obesity derived from the three definitions, while their overall prevalence rates are reduced (Figure 3). On the contrary, for younger ages, our data seem to correspond to those expected based on the literature, i.e., the IOTF definition leads to more overweight and normal weight diagnoses, while the WHO and CDC definitions detect higher obesity rates. Additionally, it seems that the overall prevalence of overweight and obesity is higher in these younger age groups, irrespective of the definition used. These observations emphasize the impact of the growth spurt that continues to take place in the first couple of years after puberty onset, and that gradually settles out in the more advanced years of adolescence.

The WHO criteria were designed for a population of children growing up in ideal conditions, whereas the other two definitions were based on populations representing various nations. It appears that when ideal conditions exist for the development of a child under 5 years of age, differences between distinct nations are smoothed out, and the final heights and weights are eventually comparable, and the differences are very small [17]. The creation of international curves for monitoring BMI based on a study of a population experiencing ideal growth conditions could potentially be useful in mitigating as many differences as possible between nations, but this may not be valid when focusing on adolescents [17]. Especially when considering the diversities existing between countries, but foremost, the impact that socio-economic conditions may have on behaviours related to energy balance, as stated in a recent survey of childhood overweight and obesity [30]. High-risk geographic areas, urbanization and demographic groups may be located by such surveys, and child/adolescent living status may be linked to obesity trends and lead to a better understanding of applying diagnostic tools such as the WHO criteria.

A limitation of the current study is the size of the population, which does not allow for separate investigations of the prevalence rates of very narrow age categories. However, the main division of the population into younger and older adolescents resulted in significant comparative observations. Additionally, our study is limited by the local environment of the region in Northwestern Greece, which influences the generalizability of the findings. Lastly, the retrospective cross-sectional design of this study did not allow for the inclusion of data on metabolic markers, access to weight interventions and follow-up outcomes, as well as data on the stage of puberty. The inclusion of such data in future studies would undoubtedly provide the opportunity for detailed associations and investigations on clinical outcomes regarding over- and underdiagnosis of overweight/obesity.

## 5. Conclusions

The application of the three international definitions in our population in Greece showed that overall and pairwise agreement was high for the three groups of normal weight, overweight and obesity. However, they lead to differences in the prevalence rates of overweight and obesity, indicating significant heterogeneity and asymmetry. The pairwise analyses demonstrated that primarily the WHO definition, and secondarily the CDC criteria, lead to a relatively augmented prevalence rate of obesity, whereas the IOTF criteria result in reduced rates of obesity, in favour of the overweight classification. Of note, puberty onset and growth spurt occurring in young adolescents (<14 years old) seem to be associated with this heterogeneity in the prevalence rates, whereas our results showed rather homogeneous classification among the definitions in older adolescents.

Caution is required when interpreting the classification resulting from the international criteria for overweight/obesity, as they cannot be used interchangeably, in particular when applying them to young adolescents. To advance precision medicine, further studies are essential for improving the classification and diagnosis of overweight/obesity by integrating subject-specific and regional characteristics.

## Figures and Tables

**Table 1:** Description of the study population.

	Total	Males	Females	*p* Value
N (%)	403	221 (54.8%)	182 (45.2%)	0.052
Age, years	12.5 (10.6–14.7)	12.5 (10.4–14.4)	12.7 (10.9–14.9)	0.062
Weight, kg	51 (40–62)	51 (39–62.5)	51 (41.7–61)	0.889
Height, cm	156 (145–165)	155 (143–169)	156 (146–162)	0.307
BMI (kg/m^2^)	20.55 (18.05–23.81)	20.26 (17.42–23.95)	20.67 (18.34–23.79)	0.494

Continuous variables are presented as median (interquartile range) with differences between groups assessed by the Mann–Whitney U test. Categorical variables are presented as count (percentage) with the difference in proportion between males/females assessed by the one-sample binomial test. BMI: Body mass index.

**Table 2:** Cross-tabulated pairwise comparison of counts according to the three different definitions in the total population.

		**CDC**			
		**Normal Weight**	**Overweight**	**Obesity**	**Total**
**WHO**	**Normal weight**	218	0	0	218
	**Overweight**	19	72	1	92
	**Obesity**	0	7	86	93
	**Total**	237	79	87	403
		**IOTF**			
**WHO**	**Normal weight**	218	0	0	218
	**Overweight**	18	74	0	92
	**Obesity**	0	31	62	93
	**Total**	236	105	62	403
		**IOTF**			
**CDC**	**Normal weight**	234	3	0	237
	**Overweight**	2	77	0	79
	**Obesity**	0	25	62	87
	**Total**	236	105	62	403

CDC: Centers for Disease Control and Prevention; WHO: World Health Organisation; IOTF: International Obesity Task Force.

**Table 3:** Cross-tabulated pairwise comparison of counts according to the three different definitions in males.

		**CDC**			
		**Normal BMI**	**Overweight**	**Obesity**	**Total**
**WHO**	**Normal BMI**	116	0	0	116
	**Overweight**	7	40	1	48
	**Obesity**	0	5	52	57
	**Total**	123	45	53	221
		**IOTF**			
**WHO**	**Normal BMI**	116	0	0	116
	**Overweight**	9	39	0	48
	**Obesity**	0	24	33	57
	**Total**	125	63	33	221
		**IOTF**			
**CDC**	**Normal BMI**	123	0	0	123
	**Overweight**	2	43	0	45
	**Obesity**	0	20	33	53
	**Total**	125	63	33	221

**Table 4:** Cross-tabulated pairwise comparison of counts according to the three different definitions in females.

		**CDC**			
		**Normal BMI**	**Overweight**	**Obesity**	**Total**
**WHO**	**Normal BMI**	102	0	0	102
	**Overweight**	12	32	0	44
	**Obesity**	0	2	34	36
	**Total**	114	34	34	182
		**IOTF**			
**WHO**	**Normal BMI**	102	0	0	102
	**Overweight**	9	35	0	44
	**Obesity**	0	7	29	36
	**Total**	111	42	29	182
		**IOTF**			
**CDC**	**Normal BMI**	111	3	0	114
	**Overweight**	0	34	0	34
	**Obesity**	0	5	29	34
	**Total**	111	42	29	182

**Table 5:** Cross-tabulated pairwise comparison of counts according to the three different definitions in younger adolescents (age < 14 years).

		**CDC**			
		**Normal BMI**	**Overweight**	**Obesity**	**Total**
**WHO**	**Normal BMI**	127	0	0	127
	**Overweight**	13	58	0	71
	**Obesity**	0	7	69	76
	**Total**	140	65	69	274
		**IOTF**			
**WHO**	**Normal BMI**	127	0	0	127
	**Overweight**	13	58	0	71
	**Obesity**	0	29	47	76
	**Total**	140	87	47	274
		**IOTF**			
**CDC**	**Normal BMI**	138	2	0	140
	**Overweight**	2	63	0	65
	**Obesity**	0	22	47	69
	**Total**	140	87	47	274

**Table 6:** Cross-tabulated pairwise comparison of counts according to the three different definitions in older adolescents (age ≥ 14 years).

		**CDC**			
		**Normal BMI**	**Overweight**	**Obesity**	**Total**
**WHO**	**Normal BMI**	91	0	0	91
	**Overweight**	6	14	1	21
	**Obesity**	0	0	17	17
	**Total**	97	14	18	129
		**IOTF**			
**WHO**	**Normal BMI**	91	0	0	91
	**Overweight**	5	16	0	21
	**Obesity**	0	2	15	17
	**Total**	96	18	15	129
		**IOTF**			
**CDC**	**Normal BMI**	96	1	0	97
	**Overweight**	0	14	0	14
	**Obesity**	0	3	15	18
	**Total**	96	18	15	129

## Data Availability

The raw data supporting the conclusions of this article will be made available by the authors upon reasonable request.

## Abbreviations

The following abbreviations/acronyms are used in this manuscript:

BMI	body mass index
WHO	World Health Organization
CDC	Centre for Disease Control and Prevention (United States)
IOTF	International Obesity Task Force

## References

[1] (2019). State of health in the eu cycle. Health at a Glance: Europe 2018.

[2] Sweatt K., Garvey W.T., Martins C. (2024). Strengths and limitations of BMI in the diagnosis of obesity: What is the path forward?. Curr. Obes. Rep..

[3] Cole T.J., Lobstein T. (2012). Extended international (IOTF) body mass index cut-offs for thinness, overweight and obesity. Pediatr. Obes..

[4] de Onis M., Onyango A.W., Borghi E., Siyam A., Nishida C., Siekmann J. (2007). Development of a WHO growth reference for school-aged children and adolescents. Bull. World Health Organ..

[5] Ogden C.L., Kuczmarski R.J., Flegal K.M., Mei Z., Guo S., Wei R., Grummer-Strawn L.M., Curtin L.R., Roche A.F., Johnson C.L. (2002). Centers for disease control and prevention 2000 growth charts for the United States: Improvements to the 1977 national center for health statistics version. Pediatrics.

[6] Phan H.D., Nguyen T.N.P., Bui P.L., Pham T.T., Doan T.V., Nguyen D.T., Van Minh H. (2020). Overweight and obesity among Vietnamese school-aged children: National prevalence estimates based on the world health organization and international obesity task force definition. PLoS ONE.

[7] Spinelli A., Buoncristiano M., Kovacs V.A., Yngve A., Spiroski I., Obreja G., Starc G., Perez N., Rito A.I., Kunesova M. (2019). Prevalence of severe obesity among primary school children in 21 European countries. Obes. Facts.

[8] Yackobovitch-Gavan M., Phillip M., Shalitin S. (2025). Comparison of who and cdc growth charts for defining weight status in the young population in Israel: A population-based cross-sectional study. Isr. J. Health Policy Res..

[9] Christoforidis A., Dimitriadou M., Papadopolou E., Stilpnopoulou D., Katzos G., Athanassiou-Metaxa M. (2011). Defining overweight and obesity among greek children living in Thessaloniki: International versus local reference standards. Hippokratia.

[10] Vidal E., Carlin E., Driul D., Tomat M., Tenore A. (2006). A comparison study of the prevalence of overweight and obese Italian preschool children using different reference standards. Eur. J. Pediatr..

[11] Sares-Jaske L., Gronqvist A., Maki P., Tolonen H., Laatikainen T. (2022). Family socioeconomic status and childhood adiposity in Europe—A scoping review. Prev. Med..

[12] Rolland-Cachera M.F. (2011). Childhood obesity: Current definitions and recommendations for their use. Int. J. Pediatr. Obes..

[13] Llorca-Colomer F., Murillo-Llorente M.T., Legidos-Garcia M.E., Palau-Ferre A., Perez-Bermejo M. (2022). Differences in classification standards for the prevalence of overweight and obesity in children. A systematic review and meta-analysis. Clin. Epidemiol..

[14] Shields M., Tremblay M.S. (2010). Canadian childhood obesity estimates based on WHO, IOTF and CDC cut-points. Int. J. Pediatr. Obes..

[15] Burla L., Knierim B., Barth J., Liewald K., Duetz M., Abel T. (2008). From text to codings: Intercoder reliability assessment in qualitative content analysis. Nurs. Res..

[16] Hayes A., Krippendorff K. (2007). Answering the call for a standard reliability measure for coding data. Commun. Methods Meas..

[17] Butte N.F., Garza C., de Onis M. (2007). Evaluation of the feasibility of international growth standards for school-aged children and adolescents. J. Nutr..

[18] Poulimeneas D., Grammatikopoulou M.G., Dimitrakopoulos L., Kotsias E., Gerothanasi D., Kiranas E.R., Tsigga M. (2016). Regional differences in the prevalence of underweight, overweight and obesity among 13-year-old adolescents in Greece. Int. J. Pediatr. Adolesc. Med..

[19] Tambalis K.D., Panagiotakos D.B., Psarra G., Sidossis L.S. (2018). Current data in Greek children indicate decreasing trends of obesity in the transition from childhood to adolescence; results from the national action for children’s health (EYZHN) program. J. Prev. Med. Hyg..

[20] Farajian P., Risvas G., Karasouli K., Pounis G.D., Kastorini C.M., Panagiotakos D.B., Zampelas A. (2011). Very high childhood obesity prevalence and low adherence rates to the mediterranean diet in greek children: The greco study. Atherosclerosis.

[21] Salehi-Abargouei A., Abdollahzad H., Bameri Z., Esmaillzadeh A. (2013). Underweight, overweight and obesity among Zaboli adolescents: A comparison between international and Iranians’ national criteria. Int. J. Prev. Med..

[22] Bahk J., Khang Y.H. (2016). Trends in measures of childhood obesity in Korea from 1998 to 2012. J. Epidemiol..

[23] Hassapidou M., Daskalou E., Tsofliou F., Tziomalos K., Paschaleri A., Pagkalos I., Tzotzas T. (2015). Prevalence of overweight and obesity in preschool children in Thessaloniki, Greece. Hormones.

[24] Kostopoulou E., Tsekoura E., Fouzas S., Gkentzi D., Jelastopulu E., Varvarigou A. (2021). Association of lifestyle factors with a high prevalence of overweight and obesity in Greek children aged 10–16 years. Acta Paediatr..

[25] Hampl S.E., Hassink S.G., Skinner A.C., Armstrong S.C., Barlow S.E., Bolling C.F., Avila Edwards K.C., Eneli I., Hamre R., Joseph M.M. (2023). Clinical practice guideline for the evaluation and treatment of children and adolescents with obesity. Pediatrics.

[26] Kassari P., Papaioannou P., Billiris A., Karanikas H., Eleftheriou S., Thireos E., Manios Y., Chrousos G.P., Charmandari E. (2018). Electronic registry for the management of childhood obesity in Greece. Eur. J. Clin. Investig..

[27] Manios Y., Vlachopapadopoulou E., Moschonis G., Karachaliou F., Psaltopoulou T., Koutsouki D., Bogdanis G., Carayanni V., Hatzakis A., Michalacos S. (2016). Utility and applicability of the “childhood obesity risk evaluation” (core)-index in predicting obesity in childhood and adolescence in Greece from early life: The “national action plan for public health”. Eur. J. Pediatr..

[28] Telford R.D., Olds T.S., Telford R.M., Cunningham R.B. (2021). The effect of height on estimates of the change in BMI-based prevalence of childhood obesity. Int. J. Obes..

[29] WHO Home/Tools and Toolkits/Growth Reference Data for 5–19 Years/Indicators/BMI-for-Age (5–19 Years) Comparison with IOTF and CDC. https://www.who.int/tools/growth-reference-data-for-5to19-years/indicators/bmi-for-age.

[30] Magriplis E., Papachatzi E., Karydas G., Chrousos G., Vantarakis A. (2025). Childhood overweight and obesity survey: An overlooked public health issue. J. Health Popul. Nutr..

